# Supplementary material for: Modeling glioblastoma heterogeneity as a dynamic network of cell states
Source: Mol Syst Biol. 2021 Sep 16;17(9):e10105. doi: 10.15252/msb.202010105 (PMC8444284; doi:10.15252/msb.202010105)
Supplement: Supplementary file 6 — Source Data for Figure 5 [file MSB-17-e10105-s004.zip › Figure5A_sourcedata/GSEA_3017/nosigdb_stateB.GseaPreranked.1622795021725/RICHARDS_NOWAKOWSKI_SCIENCE_2017_GLYC_UPREG.html]

Details for gene set RICHARDS\_NOWAKOWSKI\_SCIENCE\_2017\_GLYC\_UPREG[GSEA]

|  || Dataset | state43017 |
| Phenotype | NoPhenotypeAvailable |
| Upregulated in class | na\_neg |
| GeneSet | RICHARDS\_NOWAKOWSKI\_SCIENCE\_2017\_GLYC\_UPREG |
| Enrichment Score (ES) | -0.28250682 |
| Normalized Enrichment Score (NES) | -2.1338694 |
| Nominal p-value | 0.0 |
| FDR q-value | 0.006501461 |
| FWER p-Value | 0.018 |
Table: GSEA Results Summary

  

Fig 1: Enrichment plot: RICHARDS\_NOWAKOWSKI\_SCIENCE\_2017\_GLYC\_UPREG      
 Profile of the Running ES Score & Positions of GeneSet Members on the Rank Ordered List

  

| PROBE | GENE SYMBOL | GENE\_TITLE | RANK IN GENE LIST | RANK METRIC SCORE | RUNNING ES | CORE ENRICHMENT || 1 | IGFBP5 |  |  | 67 | 0.590 | -0.0472 | No |
| 2 | CDKN2D |  |  | 190 | 0.427 | -0.1835 | No |
| 3 | PEG10 |  |  | 206 | 0.410 | -0.1724 | No |
| 4 | H2AFZ |  |  | 216 | 0.405 | -0.1535 | No |
| 5 | TUBG1 |  |  | 268 | 0.365 | -0.1959 | No |
| 6 | TENM1 |  |  | 291 | 0.354 | -0.1990 | No |
| 7 | OLFM1 |  |  | 325 | 0.337 | -0.2186 | No |
| 8 | PARP2 |  |  | 372 | 0.319 | -0.2577 | No |
| 9 | ZNF714 |  |  | 382 | 0.313 | -0.2459 | No |
| 10 | SLC35F1 |  |  | 386 | 0.312 | -0.2258 | No |
| 11 | TMEM245 |  |  | 394 | 0.310 | -0.2114 | No |
| 12 | MPC2 |  |  | 412 | 0.303 | -0.2114 | No |
| 13 | TERF1 |  |  | 451 | 0.293 | -0.2414 | No |
| 14 | MZT1 |  |  | 456 | 0.291 | -0.2244 | No |
| 15 | EED |  |  | 459 | 0.290 | -0.2046 | No |
| 16 | HSPA4L |  |  | 461 | 0.290 | -0.1835 | No |
| 17 | NUCB2 |  |  | 533 | 0.275 | -0.2607 | Yes |
| 18 | POLR2A |  |  | 545 | 0.273 | -0.2547 | Yes |
| 19 | ALYREF |  |  | 546 | 0.273 | -0.2335 | Yes |
| 20 | CNN2 |  |  | 556 | 0.271 | -0.2250 | Yes |
| 21 | DONSON |  |  | 559 | 0.271 | -0.2067 | Yes |
| 22 | CEP57 |  |  | 605 | 0.262 | -0.2488 | Yes |
| 23 | RMDN3 |  |  | 606 | 0.262 | -0.2284 | Yes |
| 24 | PRKAR2B |  |  | 611 | 0.262 | -0.2136 | Yes |
| 25 | OXCT1 |  |  | 614 | 0.261 | -0.1961 | Yes |
| 26 | PAX6 |  |  | 617 | 0.261 | -0.1787 | Yes |
| 27 | CEP44 |  |  | 643 | 0.256 | -0.1934 | Yes |
| 28 | RPN1 |  |  | 662 | 0.254 | -0.1987 | Yes |
| 29 | CBY1 |  |  | 666 | 0.253 | -0.1833 | Yes |
| 30 | HMGCR |  |  | 683 | -0.257 | -0.1855 | Yes |
| 31 | SCD |  |  | 688 | -0.263 | -0.1706 | Yes |
| 32 | MSMO1 |  |  | 691 | -0.267 | -0.1527 | Yes |
| 33 | GDI2 |  |  | 697 | -0.274 | -0.1383 | Yes |
| 34 | STC1 |  |  | 703 | -0.279 | -0.1236 | Yes |
| 35 | NEAT1 |  |  | 714 | -0.308 | -0.1135 | Yes |
| 36 | IDI1 |  |  | 734 | -0.397 | -0.1091 | Yes |
| 37 | HMGCS1 |  |  | 739 | -0.418 | -0.0822 | Yes |
| 38 | GAP43 |  |  | 743 | -0.428 | -0.0530 | Yes |
| 39 | DDX21 |  |  | 745 | -0.440 | -0.0202 | Yes |
| 40 | SNHG3 |  |  | 749 | -0.492 | 0.0139 | Yes |
Table: GSEA details [plain text format]

  

Fig 2: RICHARDS\_NOWAKOWSKI\_SCIENCE\_2017\_GLYC\_UPREG: Random ES distribution      
 Gene set null distribution of ES for **RICHARDS\_NOWAKOWSKI\_SCIENCE\_2017\_GLYC\_UPREG**
